# Apobec2 deficiency causes mitochondrial defects and mitophagy in skeletal muscle

**DOI:** 10.1096/fj.201700493R

**Published:** 2017-11-16

**Authors:** Yusuke Sato, Hideaki Ohtsubo, Naohiro Nihei, Takane Kaneko, Yoriko Sato, Shin-Ichi Adachi, Shinji Kondo, Mako Nakamura, Wataru Mizunoya, Hiroshi Iida, Ryuichi Tatsumi, Cristina Rada, Fumiaki Yoshizawa

**Affiliations:** * Department of Agrobiology and Bioresources, Utsunomiya University, Tochigi, Japan;; † Department of Animal and Marine Bioresource Sciences, Kyushu University, Fukuoka, Japan;; ‡ United Graduate School of Agricultural Science, Tokyo University of Agriculture and Technology, Tokyo, Japan;; § Medical Research Council, Laboratory of Molecular Biology, Cambridge, United Kingdom

**Keywords:** mitochondria, myopathy, APOBEC

## Abstract

Apobec2 is a member of the activation-induced deaminase/apolipoprotein B mRNA editing enzyme catalytic polypeptide cytidine deaminase family expressed in differentiated skeletal and cardiac muscle. We previously reported that Apobec2 deficiency in mice leads to a shift in muscle fiber type, myopathy, and diminished muscle mass. However, the mechanisms of myopathy caused by Apobec2 deficiency and its physiologic functions are unclear. Here we show that, although Apobec2 localizes to the sarcomeric Z-lines in mouse tissue and cultured myotubes, the sarcomeric structure is not affected in Apobec2-deficient muscle. In contrast, electron microscopy reveals enlarged mitochondria and mitochondria engulfed by autophagic vacuoles, suggesting that Apobec2 deficiency causes mitochondrial defects leading to increased mitophagy in skeletal muscle. Indeed, Apobec2 deficiency results in increased reactive oxygen species generation and depolarized mitochondria, leading to mitophagy as a defensive response. Furthermore, the exercise capacity of *Apobec2^−/−^* mice is impaired, implying Apobec2 deficiency results in ongoing muscle dysfunction. The presence of rimmed vacuoles in myofibers from 10-mo-old mice suggests that the chronic muscle damage impairs normal autophagy. We conclude that Apobec2 deficiency causes mitochondrial defects that increase muscle mitophagy, leading to myopathy and atrophy. Our findings demonstrate that Apobec2 is required for mitochondrial homeostasis to maintain normal skeletal muscle function.—Sato, Y., Ohtsubo, H., Nihei, N., Kaneko, T., Sato, Y., Adachi, S.-I., Kondo, S., Nakamura, M., Mizunoya, W., Iida, H., Tatsumi, R., Rada, C., Yoshizawa, F. Apobec2 deficiency causes mitochondrial defects and mitophagy in skeletal muscle.

Apolipoprotein B mRNA editing enzyme catalytic polypeptide (APOBEC) family members are cytidine deaminases and have diverse roles by virtue of their ability to edit DNA and/or RNA (1–5). Apobec2 is one of the oldest members of the APOBEC family, along with the lymphoid-specific activation-induced deaminase, but its expression is restricted to differentiated cardiac and skeletal muscle in mammals and chicken (6–8). Unlike other APOBEC family members, the enzymatic activity and substrate of Apobec2 have not been fully demonstrated, and its biologic functions remain unknown (9, 10). We have previously reported that Apobec2 is decreased during denervation-induced atrophy and expressed more strongly in slow-type muscle than in fast-type muscle (11). Phenotypic analysis of Apobec2-deficient mice showed a fiber-type shift toward slow-type fiber that was accompanied by diminished body mass and muscle regeneration (evident from the accumulation of myofibers with centrally located nuclei) (12). Muscle defects associated with Apobec2 deficiency have been also documented in zebrafish by using morpholino oligonucleotide–mediated knockdown of the Apobec2 proteins (Apo2a and Apo2b), which demonstrated myopathy in the somitic musculature (evidenced by the presence of cell-free spaces, long myofibers, and impaired heart function) (13). Recent studies have suggested that Apobec2 might also play important roles in the regulation of left-right axis specification in *Xenopus* (14) and retinal regeneration of zebrafish glial cells (15–17). The exact molecular mechanism of Apobec2 action remains unclear. Some reports have hinted at a potential indirect role in controlling gene expression; for example, overexpression of Apo2b and the glycosylase MBD4 during zebrafish development has been shown to enhance genomic DNA demethylation (18), as has coexpression of human Apobec2 and the methylcytosine dioxygenase 1 TET1 in cultured mammalian cells (19). Moreover, ubiquitous overexpression of Apobec2 in transgenic mice was associated with nucleotide alterations in some transcripts in hepatocytes as well as increased liver and lung tumorigenesis (20). Thus, Apobec2 may have essential roles for body maintenance, not only in muscle but also for normal growth and development in vertebrates, despite its still questioned biochemical activity as a DNA/RNA editing enzyme.

Apobec2 is expressed in skeletal muscle and is induced during differentiation of myoblasts into fused multinucleated muscle fibers (12). Muscle fibers associate into bundles that can have different ratios of fiber types [specified by the dominant myosin heavy chain (MyHC) isoform of each fiber], thus determining the contractile properties of the muscle. Apobec2 is highly expressed in slow-type muscles, which predominantly rely on the oxidative metabolic pathway and are rich in mitochondria. Mechanical stress or biochemical defects can lead to muscle damage and/or fibrosis. Debris from damaged muscle fibers is first removed in a well-orchestrated process of phagocytosis by innate immune cells, such as neutrophils and M1- and M2-type microphages (21). The next phase of repair requires activation and recruitment of stem cells (satellite cells) that proliferate and differentiate *in situ*, leading to regeneration.

Recent observations have also linked the control of muscle regeneration to mitochondrial biogenesis and in particular to the removal of damaged mitochondria by the autophagy machinery. Furthermore, autophagy is not only vital for removing old or damaged cellular components for cellular homeostasis (22, 23); it also plays an important role in maintaining muscle mass, and its defects lead to muscle atrophy and myopathy (24–30).

In this study, we investigated the specific function of mouse Apobec2 in adult skeletal muscle. Our results demonstrate that Apobec2 deficiency causes mitochondrial defects and results in increased mitophagy (selective mitochondrial autophagy). Our findings strongly suggest that Apobec2 is required for normal mitochondrial function to maintain skeletal muscle homeostasis and identify Apobec2 as a novel congenital myopathy gene and *Apobec2^−/−^* mice as a novel animal model of myopathies of unknown origin.

## MATERIALS AND METHODS

### Mice

*Apobec2^−/−^* mice used in this study have been previously described (9). Mice were housed with free access to water and standard rodent chow. All animal experiments were performed in accordance with the Guidelines for Proper Conduct of Animal Experiments published by the Science Council of Japan and with the ethical approval of Kyushu University Institutional Review Board and regulations for the care and use of animals required by the Animal Experimentation Committee of Utsunomiya University. To monitor induced autophagy, mice were held without solid food (with free access to water) for 24 h before euthanasia and tissue collection.

### RNA extraction and real-time quantitative PCR

Expression of target and reference genes was monitored by quantitative real-time PCR with β-actin (*Actb*) or 18s rRNA used as reference. Total RNA was isolated from tibialis anterior (TA) and soleus muscles from 3 to 4 control and *Apobec2^−/−^* age-matched mice according to the Trizol-chloroform protocol (Thermo Fisher Scientific, Waltham, MA, USA). cDNA was synthesized from 1 µg of total RNA using SuperScript III (Thermo Fisher Scientific) and an oligo-dTprimer. Real-time quantitative PCR was performed using a LightCycler 1.5 (Roche, Mannheim, Germany) run under the TaqMan probe-detection protocol. Primer sets were designed according to the Roche ProbeFinder software. Primer sequences were as follows: Actb forward, CTAAGGCCAACCGTGAAAAG; Actb reverse, ACCAGAGGCATACAGGGACA; Map1lc3b forward, CCCCACCAAGATCCCAGT; Map1lc3b reverse, CGCTCATGTTCACGTGGT; Gabarapl1 forward, CATGGGCCAGCTGTATGA, Gabarapl1 reverse, CAGGTGCTCCCATCTGCT; Pink1 forward, GTCCTGAAGGGAGCAGACG; Pink1 reverse, TTAAGATGGCTTCGCTGGAG; Park2 forward, GCCCGGTGACCATGATAG; Park2 reverse GTGTCAGAATCGACCTCCACT; Ndufa5 forward, AGGGTGGTGAAGTGGAAGAG; reverse CCACCATCTGACACTGAGGT; Ndufb5 forward, TGGCAAGAGACTGTTTGTCG; reverse, CTCCCAGTGTTCAGGGATGT; Sdha forward, ACACAGACCTGGTGGAGACC; reverse, GGATGGGCTTGGAGTAATCA; Sdhb forward, TGGAACGGAGACAAG; reverse, AGCCAATGCTCGCTTC; Uqcrc1 forward, GACAACGTGACCCTCCAAGT; reverse, ACTGGTACATAGGCGCATCC; Uqcrc2 forward, AGAGGGCTTCCTGAGTG; reverse, TCGTCGAGAAAAGGCGTa; Cycs forward, GTTCAGAAGTGTGTGCCCAGTG; reverse, GTCTGCCCTTTCTCCCTTCT; Ucp3 forward, TACCCAACCTTGGCTAGACG; reverse, GTCCGAGGAGAGAGCTTGC; Ppargc1a forward, GAAAGGGCCAAACAGAGAGA; Ppargc1a reverse, GTAAATCACACGGCGCTCTT; Ppard forward, GTATGCGCATGGGACTCAC; Ppard reverse, GTCTGAGCGCAGATGGACT.

### Quantification of mitochondrial DNA by real-time PCR

Total DNA was isolated from muscles from 4 control and 4 *Apobec2^−/−^* mice using Trizol-chloroform. Copy number of mitochondrial DNA (mtDNA), *Mtco2*, was quantified by real-time quantitative PCR using a Roche LightCycler 1.5 with 18s rRNA as the control for nuclear genome DNA copy number. Primer sequences were as follows: Mtco2 forward, CCATAGGGCACCAATGATACTG; Mtco2 reverse, AGTCGGCCTGGGATGGCATC; 18S rRNA forward, CTTAGAGGGACAAGTGGCGTTC; 18S rRNA reverse, CGCTGAGCCAGTCAGTGTAG.

### Protein extraction and immunoblot analyses

Proteins were extracted from 3 to 4 control and *Apobec2^−/−^* TA muscles and homogenized in SDS sample buffer containing 125 mm Tris-HCl (pH 6.8), 5% β-mercaptoethanol, 2% SDS, and 10% glycerol. Extracted proteins were separated on acrylamide gels and then transferred onto nitrocellulose membranes (GE Healthcare, Arlington Heights, IL, USA). Blocking solution of 5% skim milk was used. An ImageQuant LAS 4000 Mini Biomolecular Imager (GE Healthcare) was used for evaluating bands. The following antibodies were used for immunoblot analysis: mouse α-tubulin (1:2000, T9026; Sigma-Aldrich, St. Louis, MO, USA), rabbit anti–light chain (LC)3B (1:1000, PM036; MBL, Nagoya, Japan), rabbit anti-p62 (1:1000, PM045; MBL), mouse FK2 anti-polyubiquitin (1:1000, Enzo Life Sciences, Farmingdale, NY, USA), rabbit anti-PINK1 (1:500, ab23707; Abcam, Cambridge, United Kingdom), rabbit anti-Parkin (1:1000, ab15954; Abcam), rabbit anti-APOBEC2 (1:1000, HPA017957; Sigma-Aldrich), mouse anti-total oxphos antibody (1:1000, ab110413; Abcam), rabbit anti-UCP3 (1:1000, ab3477; Abcam), mouse anti-Mfn1 (1:1000, ab57602; Abcam), rabbit anti-Fis1 (TTC11) (1:1000, ab96764; Abcam), mouse anti-Opa1 (1:1000, ab194830; Abcam), rabbit anti-Tom20 (1:1000, FL145; Santa Cruz Biotechnology, Dallas, TX, USA), rabbit anti-CytoC (1:1000, SAB4502234; Sigma-Aldrich), PDH-E1α (1:1000, sc65242; Santa Cruz Biotechnology), and mouse anti-BNIP3 (1:1000, ab10433; Abcam). α-Tubulin was used as a reference protein.

### Histology and immunofluorescence experiments

Extensor digitorum longus (EDL) and soleus muscles were freeze-fixed in isopentane cooled by liquid nitrogen and stored at −80°C. Fresh frozen muscle cross-sections of 5-μm thickness were stained with histochemical stains, including hematoxylin and eosin and nicotine amide adenine dinucleotide-tetrazolium reductase (NADH-TR) and modified Gomori trichrome as previously described (12). Immunohistochemical analyses were performed as follows. Briefly, frozen sections were fixed with 4% paraformaldehyde in PBS for 10 min and blocked with 3% bovine serum albumin–PBS before incubation in primary antibodies. Sections were rinsed with PBS and incubated with secondary Alexa Fluor 488– or Alexa Fluor 594–labeled goat anti-rabbit (1:500, A21441; Thermo Fisher Scientific) or chicken anti-mouse (1:500, A21201; Thermo Fisher Scientific) antibodies at room temperature for 60 min. Fluorescent images were obtained with a microscope (Eclipse 80i; Nikon, Tokyo, Japan). The following antibodies were used for immunofluorescence: rabbit anti-Apobec2 (1:100, ab51000; Abcam), mouse anti–α-actinin (1:100, ab9465; Abcam), rabbit anti-laminin (1:100, L9393; Sigma-Aldrich), rabbit anti-Tom20 (1:100, FL145; Santa Cruz Biotechnology), mouse anti-LC3B (1:100, 4E12; MBL), and mouse anti-slow myosin heavy chain (1:100, ab11083; Abcam). For quantification of LC3 intensity, the images of muscle sections were converted to black and white tiff images in ImageJ (National Institutes of Health, Bethesda, MD, USA), and the staining intensity of each fiber was measured.

### Electron microscopy analysis

For electron microscopy analysis, soleus and EDL muscle from wild-type (WT) and *Apobec2*^−/−^ mice were fixed in 3% glutaraldehyde in cacodylate buffer (pH 7.4). Specimens were postfixed in 1% osmium tetroxide in the same buffer, dehydrated with a graded series of ethanol, and embedded in epoxy resin. Ultrathin sections were stained with uranyl acetate and lead citrate and analyzed with an electron microscope (Hitachi, Tokyo, Japan).

### Measurement of metabolites

Analysis of cytosolic metabolites in TA muscles of WT and *Apobec2*^−/−^ mice were performed by C-SCOPE analysis service (Human Metabolome Technologies, Tsuruoka, Japan). Metabolites were extracted by removing protein using centrifugal filter devices after centrifugation. Extracted metabolites were analyzed by capillary electrophoresis mass spectrometry.

### Detection of reactive oxygen species and mitochondrial membrane potential

Cross-sections of soleus muscles of WT and *Apobec2*^−/−^ mice were incubated with 2 μM dihydroethidium (DHE) (D7008; Sigma-Aldrich) and DAPI in the dark for 30 min at 37°C. Red fluorescent ethidium results from the oxidation of DHE. Mitochondrial membrane potential was measured by using JC-1 dye. JC-1 is a cationic fluorescent dye that accumulates in mitochondria in a potential-dependent manner. Differentiated myotubes of WT and *Apobec2*^−/−^ mice were labeled by JC-1 (Thermo Fisher Scientific) for 30 min at 37°C. Myotubes were then imaged at both green (JC-1 monomers, low potential) and red (JC-1 aggregates, high potential). The fluorescence intensities of DHE or JC-1 aggregate areas were quantified by ImageJ.

### Measurement of exercise performance

Exercise performance was determined by allowing mice run on a treadmill until exhaustion (MK-680S; Muromachi Kikai, Tokyo, Japan). Mice were acclimated on the treadmill tilted at a 10% slope at a speed of 14 m/min for 10 min in 3 constitutive days before the test day. After acclimation, mice were run at 10 m/min for 10 min, and speed was increased by 2 m/min every 2 min until mice were exhausted. Running speed, time, and distance were recorded when the mice failed to climb the treadmill in 5 s despite the electric shock. Work and power were calculated as the following formula: work (SI) = bodyweight (kg) × gravity (9.81 m/s^2^) × vertical speed (m/s × angle) × time (s), power (W) = work (SI)/time (s).

### Statistical analysis

All data are presented as means ± sem. All assessment of significance was performed with 2-tailed Student’s *t* test, and values of *P* < 0.05 were considered significant.

## RESULTS

### Apobec2 deficiency does not alter sarcomeric structure

**Figure 1.**
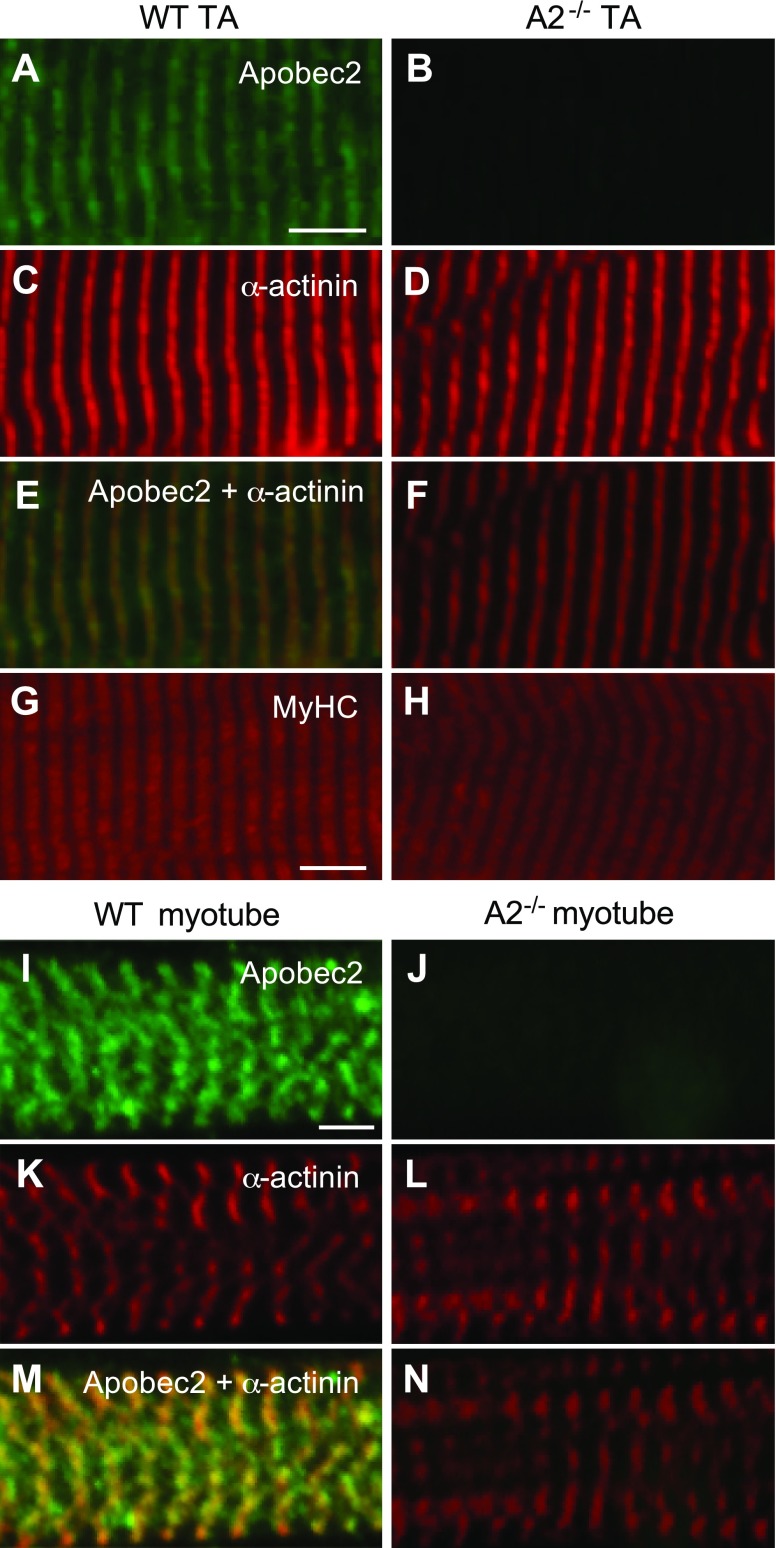
Apobec2 is localized in sarcomeric Z-lines, but its deficiency does not alter sarcomeric structure in mouse muscle. *A*–*H*) Immunostaining of longitudinal sections of TA muscle from 15-wk-old WT and *Apobec2*^−/−^ mice with antibodies against Apobec2 (*A*, *B*), α-actinin (*C*, *D*), Apobec2 + α-actinin (*E*, *F*), and MyHC (*G*, *H*). *I*–*N*) Immunostaining of primary cultured myotube from WT and *Apobec2*^−/−^ mice with antibodies against Apobec2 (*I*, *J*), α-actinin (*K*, *L*), and Apobec2 + α-actinin (*M*, *N*). *A*, *B*, *I*, *J*) Antibody specificity to Apobec2 protein. *E*, *M*) Localization of Apobec2 in Z-lines. *C*–*H*, *K*–*N*) No alteration of sarcomere structure was caused by Apobec2 deficiency. Scale bars, 5 μm.

Ectopically expressed Apobec2 is diffusely present in the cytoplasm and the nucleus in mammalian nonmuscle cell lines (31). However, the two Apobec2 homologs in zebrafish, Apo2a and Apo2b, differentially localize to the sarcomere in muscle cells, more precisely to the myoseptal boundary (13). Apo2b is also detected in the sarcomeric Z-lines of zebrafish muscle. To determine the localization of Apobec2 in mammalian muscle, we performed immunohistological analyses of longitudinal sections of the mouse TA muscle and on primary mouse myotubes isolated from hindlimb and differentiated *in vitro*. We detected specific staining for Apobec2 in the sarcomeric Z-lines (green), identified by α-actinin (red), in both mouse TA muscle and cultured myotubes (**Fig. 1*A*, *C*, *E*, *I*, *K*, *M***). Thus, in mouse skeletal muscle and in differentiated myotubes, the localization of Apobec2 was similar to that of zebrafish Apo2b (13). Morpholino oligonucleotid–mediated depletion of Apo2a or Apo2b in zebrafish results in a dystrophic phenotype with cell-free spaces in the somitic musculature and abnormal myofibers (13). Therefore, we compared the structure of the sarcomere and the basal lamina in Apobec2-deficient mice and normal control mice (Fig. 1*C–H*; Supplemental Fig. 1 related to Fig. 1). We observed normal differentiation in myoblasts isolated from the hindlimb of *Apobec2^−/−^* mice with normal fusion and development of the sarcomeric structure (Fig. 1*K*, *L*). Thus, mouse myoblasts do not require Apobec2 for the normal development of the sarcomeric structure and basal lamina.

### Mitochondrial abnormalities in *Apobec2^−/−^* skeletal muscle

**Figure 2.**
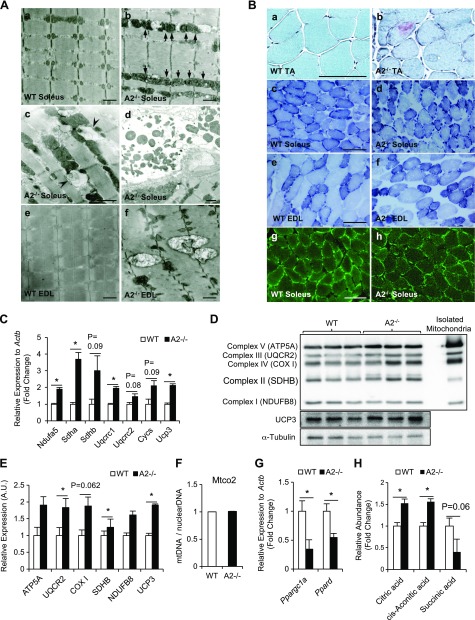
Apobec2 deficiency causes mitochondrial defects in skeletal muscle. *A*) Ultrastructural analyses of soleus (slow-type) and EDL (fast-type) muscles from 15-wk-old WT and *Apobec2*^−/−^ mice by electron microscopy. *A*) *a*, *b*) Arrows indicate elongated intermyofibrillar mitochondria with dense cristae in soleus muscle of *Apobec2^−/−^* mice; no such mitochondria were seen in WT mice. *c*, *d*) Arrowheads indicate autophagosome-like vacuoles around mitochondria (*c*) and abnormal subsarcolemmal mitochondria in soleus muscle of *Apobec2^−/−^* mice (*d*). *e*, *f*) Enlarged mitochondria in EDL muscle of *Apobec2^−/−^* mice; no such mitochondria were seen in WT mice. Scale bars, 2 μm. *B*) Histologic analyses to characterize the effects of Apobec2 deficiency on the mitochondria in skeletal muscle from 15-wk-old WT and *Apobec2*^−/−^ mice. *a*, *b*) Gomori trichrome staining of TA muscles shows damaged mitochondria by the presence of ragged red fibers in the cytosol of *Apobec2*^−/−^ mice. *c*–*f*) NADH-TR staining of soleus and EDL shows thick and dense subsarcolemmal mitochondria and less-ordered and more coarse areas in the cytoplasm of *Apobec2^−/−^*. *g*, *h*) Immunostaining with anti-TOM20 of soleus muscles shows enlarged and disordered mitochondria in *Apobec2*^−/−^ mice. Scale bars, 100 μm. *C*) Relative expressions of mitochondrial respiratory or uncoupling genes relative to *Actb* (reference gene) in soleus muscle from 15-wk-old WT and *Apobec2*^−/−^ mice. *D*) Immunoblot analysis of mitochondrial oxphos (ATP5A, UQCR2, COX I, SDHB, and NDUFB8) and UCP3 of soleus muscles from 15-wk-old WT and *Apobec2*^−/−^ mice normalized to α-tubulin. *E*) Graphs represent relative expression levels normalized to α-tubulin. *P < 0.05 (*n* = 3 for each group). *F*) mtDNA (*Mtco2*) was measured relative to *18S* rRNA as the control for nuclear genome DNA. *G*) Expression of *Ppargc1a* and *Ppard* mRNA in soleus muscle from WT and *Apobec2*^−/−^ mice relative to *Actb* (reference gene). *H*) Relative abundance of mitochondrial TCA metabolites in TA muscles from 15-wk-old WT and *Apobec2*^−/−^ mice measured by capillary electrophoresis mass spectrometry. Data are means ± sem. *P < 0.05 (*n* = 4 for each group).

Although Apobec2 deficiency did not alter the sarcomeric structure of muscle, we observed extensive phenotypic changes in the ultrastructure of the hindlimb muscles of 15-wk-old *Apobec2^−/−^* mice. Ultrastructural examination showed enlarged and/or elongated mitochondria with dense cristae in the soleus muscle compared with the small round and regular arrangement of mitochondria in control mice (**Fig. 2*A***). We also observed autophagosome-like vacuoles in intermyofibrillar spaces as well as degenerated subsarcolemmal mitochondria (Fig. 2*A*). The structurally abnormal mitochondria were not confined to slow muscle but were also present in the EDL, a predominantly fast muscle. Swollen mitochondria exceeding the size of a single sarcomere were often observed in *Apobec2^−/−^* muscle (Fig. 2*A*). Interestingly, many autophagosome-like vacuoles were observed around mitochondria, suggesting that the abnormal mitochondria associated with Apobec2 deficiency are removed by the autophagy machinery.

To characterize the effects of Apobec2 deficiency on the mitochondria in skeletal muscle, we performed histochemical analyses using the modified gomori trichrome staining, which identifies damaged mitochondria by the presence of ragged red fibers in the cytosol of individual myofibers. We identified the characteristic pattern associated with mitochondrial myopathy in sections of the TA muscle from 15-wk-old *Apobec2^−/−^* mice (Fig. 2*B*). Similarly, the NADH-TR stain showed abnormally thick and dense subsarcolemmal mitochondria and less-ordered and more coarse areas in the cytoplasm of *Apobec2^−/−^* compared with control soleus and EDL muscle fibers (Fig. 2*B*). Immunostaining of muscle cross-sections of 15-wk-old mice with the mitochondrial outer membrane marker anti-TOM20 antibody also showed enlarged and disordered mitochondria, reminiscent of the abnormal structures seen by electron microscopy in the *Apobec2^−/−^* tissues (Fig. 2*B*). The increase in size and dysmorphic mitochondria observed in *Apobec2^−/−^* tissues were accompanied by significant changes in the expression levels of mitochondrial respiratory or uncoupling genes (*Ndufa5*, *Sdha*, *Sdhb*, *Uqcrc1*, *Uqcrc2*, *Cycs*, and *Ucp3*) and an increased amount of the mitochondrial oxphose proteins (Fig. 2*C–E*). This did not reflect an increase in the total number of mitochondria because the mtDNA copy number relative to nuclear DNA was indistinguishable between control and Apobec2-deficient tissues (Fig. 2*F*). However, the transcriptional regulators of mitochondrial biogenesis, *Ppargc1α* and *Pparδ*, were significantly decreased in *Apobec2^−/−^* compared with control muscle (Fig. 2*G*). These findings are consistent with recent studies demonstrating that proliferator-activated receptor γ coactivator 1-α (PGC-1α) deficiency in muscle results in mitochondrial impairment with abnormal expression of respiratory chain enzymes but does not alter mitochondrial copy number (32–34).

To quantify the functional impact of the altered mitochondria morphology observed as a consequence of Apobec2 deficiency, we measured cytosolic metabolites using capillary electrophoresis–time-of-flight mass spectrometry in extracts from TA muscle from control and *Apobec2^−/−^* mice. Out of 116 cytosolic metabolites analyzed (Supplemental Table 1), we observed a significant increase in citric acid and *cis*-aconitic acid, both early metabolites in the tricarboxylic acid cycle (TCA), although other metabolites such as succinic acid were not significantly altered (Fig. 2*H*). This suggests that the morphologic changes associated with Apobec2 deficiency are also associated with functional impairment of mitochondria in skeletal muscle.

### Reactive oxygen species–mediated mitochondrial depolarization in Apobec2^−/−^ muscle

**Figure 3.**
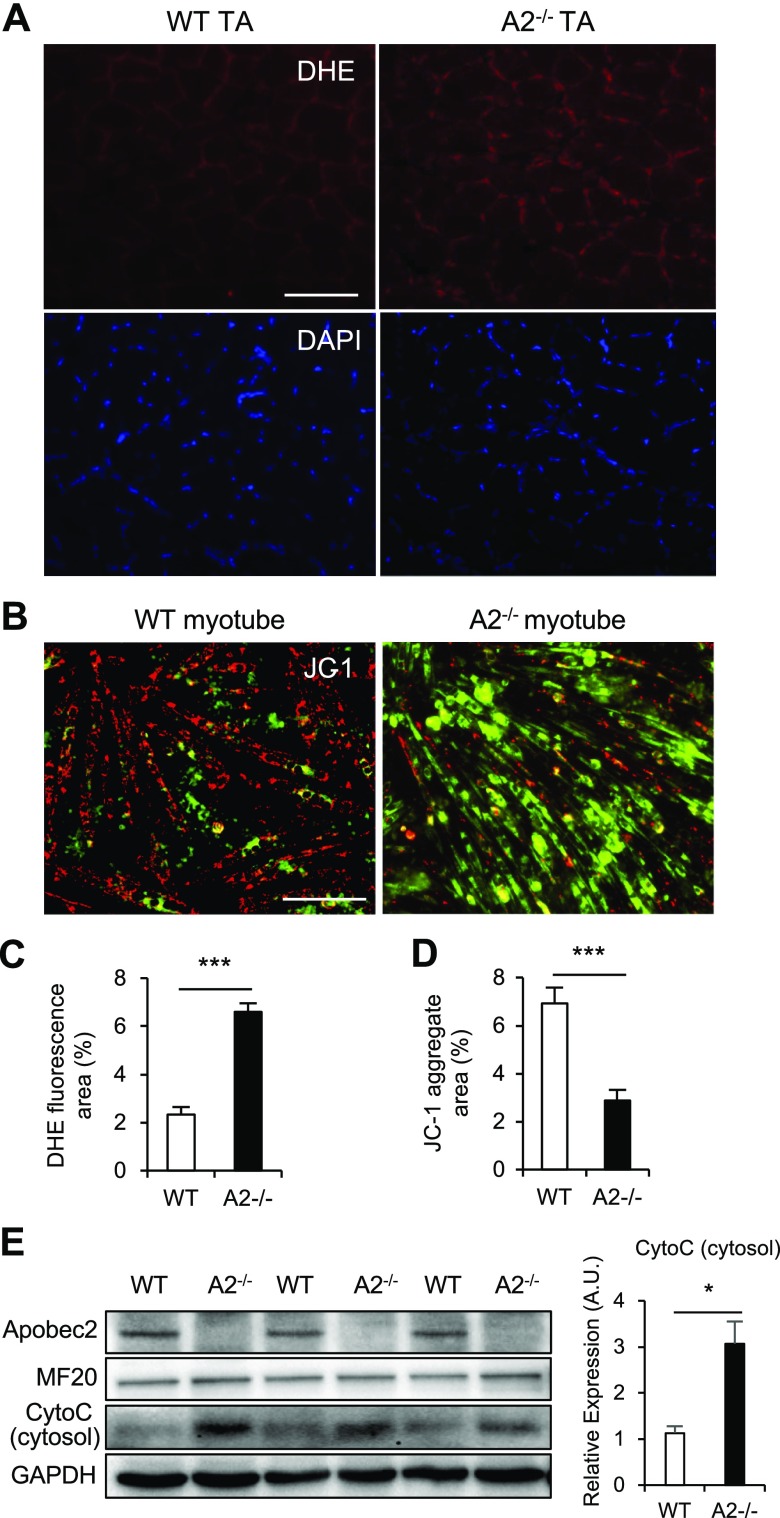
Apobec2 deficiency causes increased ROS and mitochondrial depolarization in skeletal muscle. *A*) Histologic DHE and DAPI fluorescence in TA muscles from 15-wk-old WT and *Apobec2*^−/−^ mice. Oxidized DHE in nuclei show fluorescent red. Scale bar, 100 μm. *B*) JC-1 staining of myotubes of WT and *Apobec2*^−/−^. JC-1 aggregate and monomer exhibit red (healthy) and green (depolarized) fluorescence, respectively. Scale bar, 100 μm. *C*, *D*) Graphs representing the DHE fluorescence area of *A* (*C*) and the JC-1 aggregate area of *B* (*D*). Data are means ± sem. ***P < 0.001. *E*) Immunoblot analysis of Apobec2, MF20, CytoC, and GAPDH (as an internal control) in cytosol fractionated from myotubes of WT and *Apobec2*^−/−^. Graphs represent relative CytoC expression levels normalized to GAPDH. A.U., arbitrary units. *P < 0.05.

Mitochondrial dysfunction induces reactive oxygen species (ROS) generation, leading to depolarization (35). To determine whether mitochondrial dysfunction of *Apobec2*^−/−^ muscle was caused by ROS generation, we detected the presence of ROS in transversal sections of skeletal muscle by using DHE. Indeed, we observed an increased DHE fluorescence in *Apobec2*^−/−^ muscle (**Fig. 3*A*, *C***). ROS can induce rapid depolarization of inner mitochondrial membrane potential and subsequent impairment of function (35). Therefore, we analyzed the mitochondrial membrane potential of differentiated myotubes of WT and *Apobec2*^−/−^ by fluorescence staining with JC-1. JC-1 forms an aggregate in polarized mitochondria that results in red fluorescence. However, the JC-1 monomer present in cells with depolarized mitochondrial membranes results in green fluorescence. Quantification of the red fluorescent intensities indicates that *Apobec2*^−/−^ myotubes were depolarized compared with WT (Fig. 3*B*, *D*). Increased cytosolic cytochrome c protein in *Apobec2*^−/−^ myotubes also supports the presence of mitochondrial depolarization and ROS generation (Fig. 3*E*).

### Mitochondrial dysfunction leads to increased mitophagy in *Apobec2^−/−^* muscle

**Figure 4.**
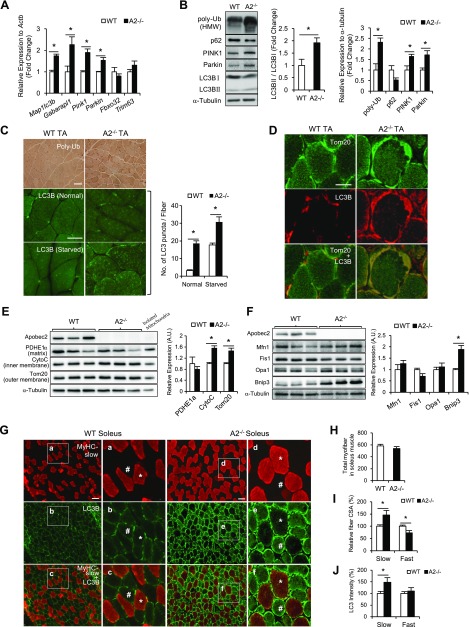
Mitophagy is enhanced in *Apobec2^−/−^* muscle. *A*) Expressions of autophagy-related genes in TA muscles from 15-wk-old WT and *Apobec2*^−/−^ mice (*n* = 4 for each group) relative to *Actb* mRNA expression as reference gene. Graphs represent means ± sem. *P < 0.05. *B*) Immunoblot analysis of poly-Ub, p62, PINK1, Parkin, and LC3B (LC3B-II/LC3B-I) in TA muscles from 15-wk-old WT and *Apobec2*^−/−^ mice. Graphs represent LC3B-II/LC3B-I ratios, poly-Ub, p62, PINK1, and Parkin levels normalized to α-tubulin. Bands are representative images of 3 independent experiments. HMW, high molecular weight. *P < 0.05. *C*) Immunostaining of cross-sections of TA muscle from 15-wk-old C57Bl/6 and *Apobec2*^−/−^ mice, with anti–poly-Ub (top) and anti-LC3B in normal mice (middle) or mice held without food overnight (bottom). Numbers of LC3B-positive puncta quantified from 50 randomly selected myofibers are summarized as histograms. *D*) Immunostaining with anti-Tom20 and anti-LC3B of cross-sections of TA muscle from a 15-wk-old WT mouse and an *Apobec2*^−/−^ mouse. Colocalization of LC3B-positive puncta with Tom20 suggests mitophagy in the *Apobec2^−/−^* mouse. *E*) Immunoblot analysis of PDHE1α, CytoC, and Tom20 in soleus muscles from 15-wk-old WT and *Apobec2*^−/−^ mice. Graphs represent relative expression levels normalized to α-tubulin. *P < 0.05 (*n* = 3 for each group). *F*) Immunoblot analysis of Mfn1, Fis1, Opa1, and BNIP3 in soleus muscles from 15-wk-old WT and *Apobec2*^−/−^ mice. Graphs represent relative expression levels normalized to α-tubulin. *P < 0.05 (*n* = 3 for each group). *G*) Immunostaining with MyHC-slow (red) and LC3B (green) of cross-sections of soleus muscle (slow-type) from 15-wk-old WT and *Apobec2*^−/−^ mice. Insets show examples of slow (*) and fast (#) myofibers. *H*) Total number of fibers per field in soleus muscle (data from 4 individual mice). *I*) Average cross-sectional area (CSA) of MyHC-slow positive (red) and other myofibers of MyHC-slow negative mice (*n* = 4). *J*) Relative intensity of anti-LC3B staining in muscle sections according to MyHC type.

Dysfunctional, depolarized mitochondria can produce higher amounts of ROS, leading to the release of cytochrome c and subsequent apoptosis. Therefore, ROS-producing, depolarized mitochondria are the substrate for mitophagy (36, 37). We investigated whether the defects associated with Apobec2 deficiency—in particular the altered mitochondria—were associated with changes in the basal levels of autophagy and mitophagy. The expression levels of autophagy/mitophagy-related genes in TA muscle, monitored by quantitative PCR, were significantly altered in *Apobec2^−/−^* samples, with elevated values for *Map1lc3b* and *Gabarapl1* as well as *Pink1* and *Parkin* (Fig. 4*A*). Thus, both the general autophagosome formation and the mitophagy pathways are induced in Apobec2-deficient muscle. The ubiquitin ligases *Trim63* and *Fbxo32*, which are associated with inflammation-induced muscle atrophy and cachexia, remained within normal levels, suggesting that the induction of the autophagocytic pathway was specific to mitochondria rather than a general increase in the catabolic pathway (**Fig. 4*A***). The hyperactivation of the autophagy pathway was also evident by the increased levels of LC3B as well as general poly-ubiquitinated proteins (Fig. 4*B*) (38). Furthermore, we observed an increased in the ratio of LC3B-II/LC3B-I proteins in *Apobec2^−/−^* muscle, an indication of enhanced autophagosome maturation (Fig. 4*B*). In addition, we observed decreased levels of p62 protein, an adaptor linking ubiquitinated proteins with the autophagic machinery, suggesting that the enhancement in autophagy was accompanied by increased autophagosome degradation (Fig. 4*B*).

Phosphatase and tensin homolog–induced putative kinase 1 (PINK1) and the E3 ubiquitin ligase Parkin (PARK2) are known regulators of mitophagy, promoting the specific ubiquitination of mitochondria and promoting their degradation (39, 40). Both proteins were elevated in Apobec2-deficient muscle (Fig. 4*B*), confirming that the hyperactivation of the autophagy pathway was specifically directed to enhance mitophagy, although we detected no alteration in the phosphorylation levels of mechanistic target of rapamycin (mTOR), the main regulatory pathways of autophagy (Supplemental Fig. 2 related to Fig. 4*B*).

In agreement with the increased global levels of poly-ubiquitinated proteins observed in tissue extracts from Apobec2-deficient muscle, we observed increased poly-ubiquitin signal in individual myofibers by immunohistochemistry compared with controls (Fig. 4*C*, top panel). Similarly, immunostaining with anti-LC3B to identify autophagosomes detected increased LC3B fluorescent puncta in *Apobec2^−/−^* TA muscle. Although LC3B puncta were induced after starvation and appeared distributed throughout the myofibers in both control and Apobec2-deficient muscle, puncta were significantly higher in *Apobec2^−/−^* mice (Fig. 4*C*), indicating that the elevated basal level of autophagy was further induced. Immunostaining of transversal section of soleus muscle also demonstrated the colocalization of LC3B with mitochondria, identified by the anti-TOM20 staining, including the subsarcolemmal mitochondria observed in *Apobec2^−/−^* tissues (Fig. 4*D*).

PINK1 promotes Parkin-mediated mitophagy by recruiting Parkin to mitochondria and is localized on the mitochondrial outer membrane when mitochondria are defective (41). Because we observed increased levels of mitochondrial inner/outer membrane proteins in *Apobec2^−/−^* soleus muscle (Tom20 and CytoC) (Fig. 4*E*), we next measured mitochondrial inner/outer membrane localized proteins Mfn1, Fis1, Opa1, and BNIP3, which related to mitochondrial fission, fusion, and mitophagy (42, 43). Although there was no alteration in Mfn1, Fis1, and Opa1 expression, BNIP3 (BCL2/adenovirus E1B 19-kDa interacting protein 3), known as mitophagy receptor (44–46), was increased in *Apobec2^−/−^* soleus muscle, indicating the enhanced mitophagy without increased mitochondrial fission and fusion (Fig. 4*F*). These results demonstrate that mitophagy is highly induced in skeletal muscle as a result of Apobec2 deficiency.

Although Apobec2 is expressed at higher levels in slow-type muscle than in fast-type muscle (12), it is unclear to what extent the muscle atrophy associated with Apobec2 deficiency affects different fiber types. Whereas the overall number of myofibers in *Apobec2^−/−^* soleus muscle was unaltered, we detected swollen and significantly larger slow-type myofibers (slow-MyHC positive) with the characteristic thickened subsarcolemmal mitochondria (Fig. 4*G*, *H*). Quantification of average area per fiber also showed that, whereas the slow fibers were larger than in control muscle, the MyHC-slow negative (fast-type MyHC) fibers were significantly smaller (Fig. 4*I*). These size differences were consistent with our previous biochemical quantification of the relative amount of fast- *vs.* slow-type MyHC in soleus muscle, which indicated a specific deficit of type II MyHC and an increase in type I MyHC (12). Interestingly, MyHC-slow negative myofibers also had stronger signals of autophagosomal membrane protein LC3B, although MyHC-slow positive and negative myofibers in WT muscle had broad signals of LC3B (Fig. 4*J*). Overall these results suggest that Apobec2 deficiency leads to differential atrophy of fast-type fibers.

**Figure 5.**
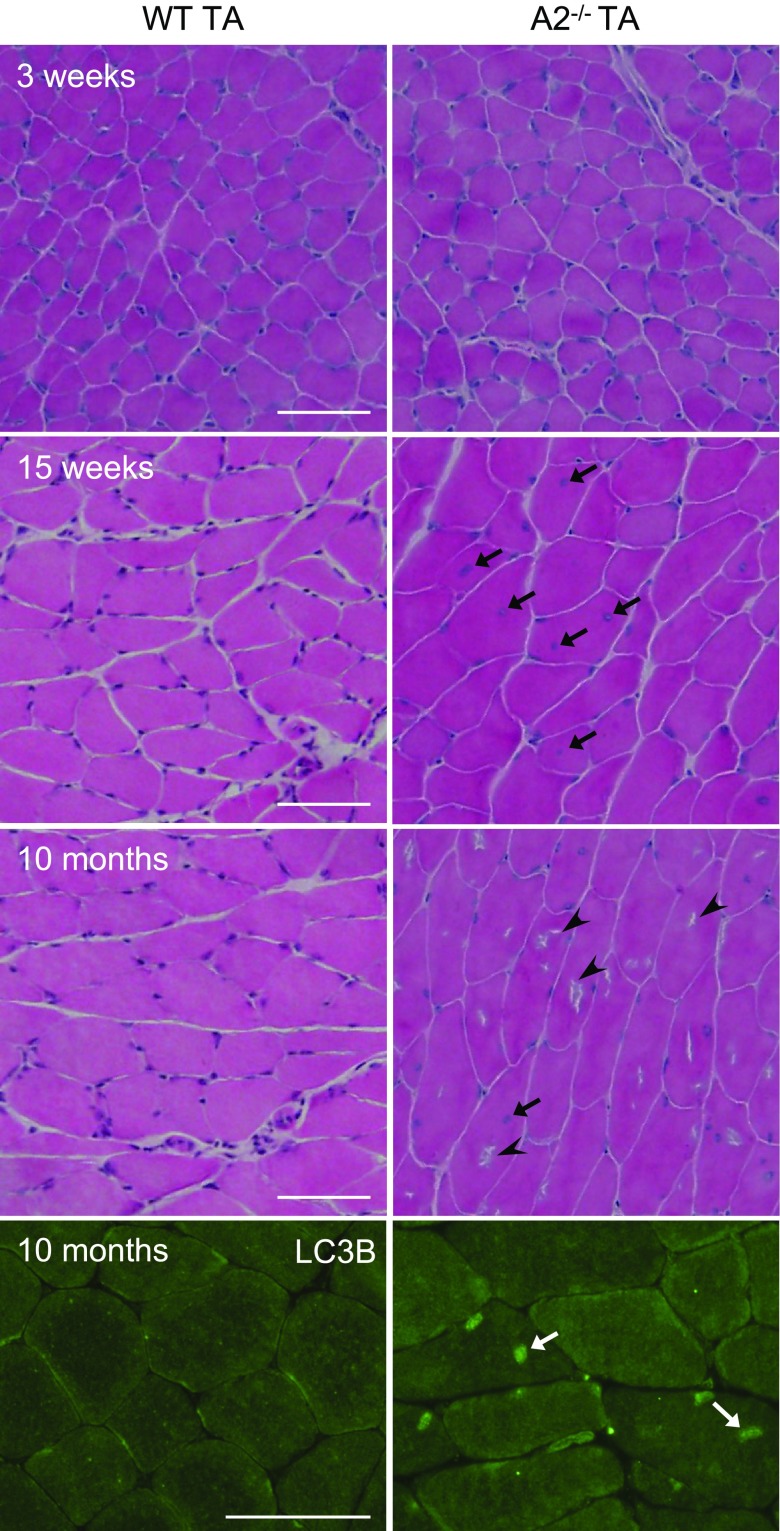
Myofibers of *Apobec2^−/−^* mice accumulate rimmed vacuoles in old age. Hematoxylin and eosin staining of cross-sections of TA muscles from 3-wk-old, 15-wk-old, and 10-mo-old WT and *Apobec2*^−/−^ muscle. Arrows indicate centrally located nuclei in 15-wk-old­ and 10-mo-old *Apobec2*^−/−^ myofibers. Arrowheads indicate rimmed vacuoles in 10-mo-old *Apobec2*^−/−^ myofibers. Immunostaining of 10-mo-old muscles with LC3B antibody shows positive structures in *Apobec2*^−/−^ myofibers (white arrow). Scale bars, 100 μm.

The early-onset phenotypic changes observed in *Apobec2^−/−^* muscle are clinical signs of myopathy, including centrally located nuclei, irregular fiber size, and small myotubes (12). We also characterized late-onset phenotypic changes in older animals. We observe extensive rimmed vacuoles in hematoxylin–eosin sections (**Fig. 5**) in 10-mo-old *Apobec2^−/−^* mice. Rimmed vacuoles are frequently observed in myopathies associated with autophagy and lysosomal diseases (47, 48). Observed vacuoles in *Apobec2^−/−^* mice were stained with LC3B antibody, suggesting the pathologies were associated with autophagy dysfunction. Thus, excessive or defective autophagy and lysosome-associated pathologies can affect the maintenance of muscle mass and homeostasis (24–30). The late-onset phenotype observed in *Apobec2^−/−^* mice would suggest that the increase in autophagy capacity associated with age is exacerbated by the chronic defects in the mitochondria, eventually tilting the balance against remodeling.

### Functional myopathy in *Apobec2^−/−^* mice

**Figure 6.**
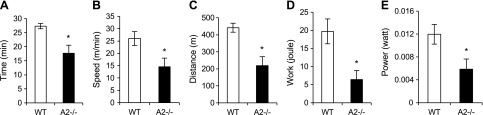
Impaired exercise capacity in *Apobec2^−/−^* mice. Treadmill exercise was performed in 15-wk-old WT and *Apobec2*^−/−^ mice after 3 d of acclimation. Mice ran at speed with a 2-m increase per minute at 10% slope until they failed to climb the treadmill in 5 s despite negative reward (electric shock). Time (*A*), speed (*B*), and distance (*C*) were recorded. Work (*D*) and power (*E*) were calculated with the normalization of individual body weight. All graphs represent means ± se. *P < 0.05 (*n* = 4 for each group).

Given the mitochondrial dysfunction associated with Apobec2 deficiency, we tested the physiologic consequences on the overall muscle function under stress conditions. We exposed mice to increased exercise load on a treadmill test with progressive speed and quantified the time until exhaustion. *Apobec2^−/−^* mice remained active for shorter periods (35% less time than control mice), and they reached maximal speed at 15 m/min, compared with 26 m/min for control mice (**Fig. 6*A*, *B***). This indicated that, on average, the running distance was decreased by 50% in *Apobec2^−/−^* mice (Fig. 6*C*). Parameters of work and power calculated based on body weight and treadmill slope also showed 67 and 51% decrease, respectively, compared with control mice (Fig. 6*D, E*). These results demonstrated a dramatic impairment in the exercise capacity of *Apobec2^−/−^* mice, confirming that the mitochondrial functional impairment associated with the lack of Apobec2 leads to muscular dysfunction.

## DISCUSSION

Although the zinc-coordinating core of Apobec2 is highly conserved and structurally close to other activation-induced deaminase/APOBEC family members (49), the catalytic activity of Apobec2 remains controversial, with no consensus on either its substrates or its physiologic and molecular functions (9, 10). In this study, we describe a physiologic function of Apobec2 in maintaining mitochondrial homeostasis in skeletal muscle.

Apobec2 has been implicated in DNA demethylation (18), a process essential for normal myoblast differentiation and fusion into myotubes (50), as well as for normal development in zebrafish and *Xenopus* (14–17). Our results confirmed the localization of mouse Apobec2 in the sarcomeric Z-lines in adult muscle tissue and in primary cultured myotubes, as has been reported for zebrafish Apob2b (13). Although induced in response to myogenic differentiation signals (12), our results further confirmed that Apobec2 is not required for myogenic fusion or differentiation. Because the sarcomere structure is not affected in *Apobec2^−/−^* muscle, we considered that Apobec2 does not contribute to the maintenance of sarcomere structure even though it was located at Z-line. Many proteins, not only those important for maintenance of sarcomere structure but also metabolic enzymes and signaling molecules, are located at the Z-line of muscle cells (51). Because Apobec2 has only detected in cytosolic fraction of rat skeletal muscles (11), its function may be associated with mitochondrial homeostasis in cytosol.

In mice, Apobec2 is clearly not essential during development, but muscle-specific defects (atrophy) are already present at birth, suggesting an intrinsic defect in terminally differentiated muscle that becomes aggravated with exercise and age (12). Skeletal muscle atrophy is a genetically controlled proteolysis process involving the activation of the autophagy lysosome and the ubiquitin–proteasome system, which control not only protein turnover and the size of the myofibers but also cellular homeostasis. Although we found expression of the ubiquitin ligases (*Fbxo32* and *Trim63*) and the phosphorylation levels of mTOR, a main regulatory pathway of proteolysis, unaltered by Apobec2 deficiency, we observed multiple autophagic vacuoles around mitochondria and elongated or enlarged mitochondria as well as mitochondria containing abundant cristae, which has been described as a defensive response to exacerbated autophagy (52). Autophagy and mitophagy are important not only for organelle degradation and mitochondrial health but also for maintenance of skeletal muscle mass (24, 25, 53), as exemplified by transgenic mice where autophagy activation or inhibition results in muscle atrophy (24, 49, 54). Impaired autophagy or mitophagy are known to promote the accumulation of nuclear abnormalities, reduce cell viability, and induce muscle atrophy (27, 28) and have been implicated in various human diseases, including cancer, cardiac/skeletal muscle disease with atrophy, and metabolic disorders (55, 56). Indeed, skeletal muscle from autophagy-deficient mice closely mimics muscle from patients with sarcopenia and myopathy (47). Thus, the enhanced mitochondrial autophagy (mitophagy) observed in *Apobec2^−/−^* muscle likely resulted from mitochondrial defects.

Dysmorphic mitochondria and thickened subsarcolemmal mitochondria were evident in tissue sections from *Apobec2^−/−^* muscle and were accompanied by up-regulation of mitochondrial respiratory protein expression and increased ROS generation. Mitochondrial respiratory chains transport protons and create the electrochemical proton gradient across the inner mitochondrial membrane to synthesize ATP. ROS are generated during these aerobic metabolism processes and can lead to oxidative damage of mitochondrial proteins and DNA, impairing the ability of mitochondria to synthesize ATP and metabolites, including the TCA cycle. The mitochondrial TCA metabolites citric acid and *cis*-aconitic acid were significantly increased in Apobec2-deficient tissue extracts. However, there were no significant differences in 2-oxoglutaric acid and acetyl-CoA, both mitochondrial TCA metabolites implicated in the regulation of autophagy by activating the mTOR pathway (57, 58). Consequently, the phosphorylation levels of mTOR were not altered in *Apobec2^−/−^* muscle. However, we documented the decrease in transcriptional regulators of mitochondrial biogenesis, *Ppargc1α* and *Pparδ* in *Apobec2^−/−^*, suggesting that the altered TCA metabolites were likely the result of impaired mitochondrial function. PGC-1α deficiency in muscle causes mitochondrial impairment and abnormal expression of respiratory chain enzymes but does not alter mitochondrial copy number (32–34). We also observed no alteration of the mtDNA copy number in Apobec2-deficient tissue, but presumably this reflected compensatory regeneration of mitochondria.

It has been demonstrated that mitochondrial depolarization is the consequence of a decrease in the transmembrane potential. Dysfunctional, depolarized mitochondria can produce higher amounts of ROS, leading to the release of cytochrome c and subsequent apoptosis. Previous studies demonstrate that mitochondrial depolarization precedes mitophagy as a defensive response (35). Indeed, we observed increased expression of BNIP3, a receptor for mitophagy, as a defensive response against defective mitochondria (44, 45) because BNIP3 overexpression induces autophagy in skeletal muscle *in vivo* (53). Expression of *Bnip3* in *Drosophila* also suppresses muscle degeneration and the mitochondrial abnormality caused by *PINK1* inactivation (46).

Defective autophagy plays a role in congenital muscular dystrophies caused by defects in collagen VI, laminin A/C, and dystrophin (59–61), but myopathy can also be the result of alterations in mitophagy-related genes such as Chkb and Mul1 (25, 26). These mice display defects in mitochondria, muscle weakness, and muscle atrophy, but the exact mechanisms are still not defined.

We searched DisGeNET v5.0 (*http://www.disgenet.org/web/DisGeNET/menu/search*; a public database from genome-wide association studies) to find Apobec2-associated myopathy, but the search result revealed no apparent association of Apobec2 with any myopathy so far examined. However, our results could be interpreted as suggesting that Apobec2 acts as an unidentified autophagy/mitophagy-related gene.

In summary, this study shows that mouse Apobec2 deficiency causes mitochondrial defects in skeletal muscle and induced mitophagy, both resulting in myopathy and muscle atrophy. Thus, *Apobec2^−/−^* mice provide a novel mammalian model for understanding mitophagy in muscle regeneration and its links to myopathy.

## Supplementary Material

This article includes supplemental data. Please visit *http://www.fasebj.org* to obtain this information.

## Abbreviations

APOBEC: apolipoprotein B mRNA editing enzyme catalytic polypeptide
DHE: dihydroethidium
EDL: extensor digitorum longus
LC: light chain
mtDNA: mitochondrial DNA
MyHC: myosin heavy chain
mTOR: mechanistic target of rapamycin
NADH-TR: nicotinamide adenine dinucleotide tetrazolium reductase
PGC-1α: proliferator-activated receptor γ coactivator 1-α
PINK1: phosphatase and tensin homolog–induced putative kinase 1
TCA: tricarboxylic acid cycle
ROS: reactive oxygen species
TA: tibialis anterior
WT: wild type
