# Supplementary material for: Apobec2 deficiency causes mitochondrial defects and mitophagy in skeletal muscle
Source: FASEB J. 2017 Nov 16;32(3):1428–39. doi: 10.1096/fj.201700493R (PMC5892721; doi:10.1096/fj.201700493R)
Supplement: Supplementary file 1 [file fj.201700493R.sd1.docx]

**Table S1. List of metabolites measured by CE-TOF-MS.**

Cytosolic metabolites in TA muscles of WT and A2^-/-^ were measured by CE-TOF-MS. *P* values less than 0.05 were considered significant and assessment of significance was performed with Welch t-test.

**Figure S1, related to Figure 1. No** **alteration in the protein level of Laminin by** **Apobec2 deficiency.**

Immunoblot analysis of Laminin in TA muscle from 15-week-old WT and *Apobec2^-/-^* mice normalized to GAPDH. Graph represents mean ± SEM. Cross sections of TA muscle stained with anti-laminin (green). Scale bar = 50 μm.

**Figure S2, related to Figure 3. No** **alteration in the phosphorylation levels of mTOR by** **Apobec2 deficiency.**

Immunoblot analysis of phosphorylated-mTOR (p-mTOR) and total mTOR in TA muscle from 15-week-old WT and *Apobec2^-/-^* mice. Graph represents mean ± SEM.
